# Petition to the Municipal Council of Paris, France

**Published:** 1882-07

**Authors:** 


### PETITION TO THE MUNICIPAL COUNCIL OF PARIS, FRANCE.

President of the Municipal Council:

The undersigned, elector of the Second District of Paris, living at 40 Rue Sainte Anne, thinks it his duty to call your attention and that of your colleagues, to an important matter of public health. A point absolutely neglected by the most numerous of the population, *i.e.*, the working classes. In all the lyceums, colleges and private schools of both sexes, a dentist is attached to each institution, and makes periodical visits for the care of the mouth. He also directs the treatment during the first and second dentition—a critical time, particularly from the dentists' standpoint, and one upon which the general health of the person depends in after life. Physical deformity, severe periodical pains in the face, rapid decay, contusions, disorders of the digestive organs are some of the serious consequences.

The poverty, the ignorance of the elementary principles of dental hygiene, many absurd prejudices, the silence of children fearing any painful operations, all conspire to make the younger generation lose the benefits arising from careful periodical attention. This state of neglect is followed by entire physical degeneracy, for which it is the duty of our officers to find some remedy which shall diminish the causes. The great importance of watchful care and direction in the dentition of children is not to be demonstrated here before a jury as competent as the Municipal Council of Paris. To call your attention to this question is to be assured of a favorable solution in preparing a project of organization at once practical and gratuitous, for dental service in the primary schools of Paris." E. Taillebois.

This petition, supplemented by one presented and signed by the members of the two leading dental societies of Paris has met with the favorable response desired, and now the dental services rendered the more indigent classes of Paris, under the supervision of municipal authority, is to be the means of inciting other cities and countries to the same beneficial arrangement. Petitions desiring the same result have been prepared in the large cities of Spain, Italy, England, and other countries. A letter from M. Taillebois, some time ago, announcing the result of what has been a long, persistent effort on the part of the dentists of Paris, says that sufficient time has elapsed to show how well appreciated and wise this law is. In our own country there is only needed a well-concerted effort by the dentists of each city to accomplish what has been done in Paris.

The appointment of dentists, who shall make periodical examination and give careful attention to the needs of the children in our schools, is one that should be favorably received by all. A few years, only, would show how valuable this attention to such important organs as the teeth would be in the early education of children of all classes. There is in nothing else relating to the hygiene of the body such culpable ignorance shown as in the care of the teeth, and those of children especially. Miseries in after life, resulting from the neglect of these organs, would then be more clearly traceable to the true cause. The proper education of all classes is needed in this respect; but this one class—the poor—has been such sufferers from these causes that there should be an earnest effort made to give them the relief needed, and this beginning in our schools will be an important step in the right direction.

